# Multi-purpose ECG telemetry system

**DOI:** 10.1186/s12938-017-0371-6

**Published:** 2017-06-19

**Authors:** Matteo Marouf, Goran Vukomanovic, Lazar Saranovac, Miroslav Bozic

**Affiliations:** 1 School of Electrical Engineering, University of Belgrade, Bulevar kralja Aleksandra 73, 11120 Belgrade, Serbia; 2 University Children’s Hospital, Tirsova 10, 11000 Belgrade, Serbia; 3 Faculty of Electronic Engineering, University of Nis, Aleksandra Medvedeva 14, 18000 Nis, Serbia

**Keywords:** Telemetry systems, ECG, Validation, Dry electrodes, Event recording, Post-event

## Abstract

**Background:**

The Electrocardiogram ECG is one of the most important non-invasive tools for cardiac diseases diagnosis. Taking advantage of the developed telecommunication infrastructure, several approaches that address the development of telemetry cardiac devices were introduced recently. Telemetry ECG devices allow easy and fast ECG monitoring of patients with suspected cardiac issues. Choosing the right device with the desired working mode, signal quality, and the device cost are still the main obstacles to massive usage of these devices.

**Methods:**

In this paper, we introduce design, implementation, and validation of a multi-purpose telemetry system for recording, transmission, and interpretation of ECG signals in different recording modes. The system consists of an ECG device, a cloud-based analysis pipeline, and accompanied mobile applications for physicians and patients. The proposed ECG device’s mechanical design allows laypersons to easily record post-event short-term ECG signals, using dry electrodes without any preparation. Moreover, patients can use the device to record long-term signals in loop and holter modes, using wet electrodes. In order to overcome the problem of signal quality fluctuation due to using different electrodes types and different placements on subject’s chest, customized ECG signal processing and interpretation pipeline is presented for each working mode.

**Results:**

We present the evaluation of the novel short-term recorder design. Recording of an ECG signal was performed for 391 patients using a standard 12-leads golden standard ECG and the proposed patient-activated short-term post-event recorder. In the validation phase, a sample of validation signals followed peer review process wherein two experts annotated the signals in terms of signal acceptability for diagnosis.We found that 96% of signals allow detecting arrhythmia and other signal’s abnormal changes. Additionally, we compared and presented the correlation coefficient and the automatic QRS delineation results of both short-term post-event recorder and 12-leads golden standard ECG recorder.

**Conclusions:**

The proposed multi-purpose ECG device allows physicians to choose the working mode of the same device according to the patient status. The proposed device was designed to allow patients to manage the technical requirements of both working modes. Post-event short-term ECG recording using the proposed design provide physicians reliable three ECG leads with direct symptom-rhythm correlation.

## Background

Over the last few years, many ECG measuring applications emerged taking advantage of the widespread use of smart phones. Patients with cardiac issues, as well as healthy people, can now record ECG signals and send them to physicians or health centers using developing communication technology, which helps to enable ECG recording regardless of place and time. Different designs of ECG devices were proposed to operate in telemedicine system in order to make the procedure of signal recording easy and smooth for users [1–5].

Generally, personal ECG devices could be divided into holter devices, and event recorders. Holter signal is an ECG recording done over a period of 1–7 days, wherein three electrodes, at least, are attached to the patient’s chest and connected to a small portable ECG recorder, generally by lead wires [6]. Patients keep a diary of their symptoms and function normally with their daily activities, with the exceptions of activities such as taking a shower, swimming, or any activity causing an excessive amount of sweating, that cause the electrodes to become loose or detached during recording [6].

The main limitation of Holter monitoring is the detection of intermittent arrhythmias, because symptoms happen infrequently. Additionally, there is no real-time analysis of the recoded signals. In these cases, event monitor could be used [6–9].

The second type of ECG monitoring applications is the event monitoring. Event recording devices can be divided into loop and post-event recorders. In loop recording approach, electrodes are in long-term continuous contact with patient’s skin and the event signal storing and processing is triggered by patients or by embedded algorithm [10, 11].

Different devices emerged to make the loop ECG event re-coding easier and wireless [2–4] using wearable fashion such as belts and T-shirts. However, the quality of the recorded signals is still the major impediment facing the efforts to replace signals recorded with standard wet adhesive electrodes which are still the favored choice for long-term recording [12]. Poor signal quality and, consequently, poor clinical acceptability are the main reason for imprecise delineation and misclassification of heart beats with artifacts. Moreover, the lack of signal quality makes the algorithm event-activated devices generate false alarms and store misleading intervals which increase the physician cost [6].

The second type of event monitoring is the patient-activated post-event ECG recording where the device is not worn continuously, but applied and triggered by patients once symptoms develop [7, 13, 14]. Event ECG intervals are then recorded and transmitted directly to a data center where signals can be processed and analyzed by both algorithms and physicians.

We propose multi-purpose ECG device and a telemetry system platform wherein the device is operating. Both long-term holter and post-event short-term recording modes are enabled using single device. The design and implementation of the proposed device and processing pipeline make these different ECG recording modes smooth and easy to be done by a layperson.

In this work we briefly describe the system design and architecture. We show the evaluation process and validation results, and finally, conclusion is drawn.

## System architecture and design

**Fig. 1:**
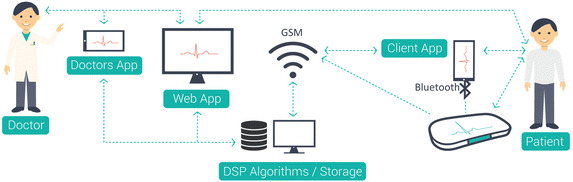
Shows the basic diagram of the presented platform and its principal components, where the proposed device is wirelessly transmitting the recordings to a handheld mobile phone which transmits the signals to a cloud server. Signals could be directly transmitted to the cloud server using GSM connection

The basic scheme of the telemedicine system in which the ECG device is supposed to work is shown in Fig. 1. The system consists of three main components: an ECG device, an algorithms/storage server, and users’ applications for signal recording, transmission, and cloud-based analyses. The basic concept is to allow patients to record and send ECG signals to the algorithms/storage centre. Experts have instant access to the sent signals using mobile and web applications where they can view all sent signals and algorithm’s proposals for them.

Recorded signal is sent from the ECG device to algorithms/storage either via Bluetooth to phone application which will send them to algorithm/storage server using the phone GSM network Internet service, or directly via GSM/GPRS module embedded in the device that communicates directly with the server using the GSM operator network. The last option is important, especially for patients who don’t use smart phones, such as parts of the elderly population, and for fast instant ECG signal transmission when a smart phone is not operable.

Received signals are further processed on the server and then classified into critical or urgent and uncritical signals. Urgent signals are signals sent with an urgent flag by patients or those include rhythm that is not considered as normal rhythm by algorithms. Thus, experts receive a notification when any signal is received and an urgent notification when the signal is flagged as urgent. Processing of signals and their classification into urgent and uncritical helps to reduce the workload of physicians and reduces the cost of the whole telemedicine platform.

### Mechanical design and working modes

**Fig. 2:**
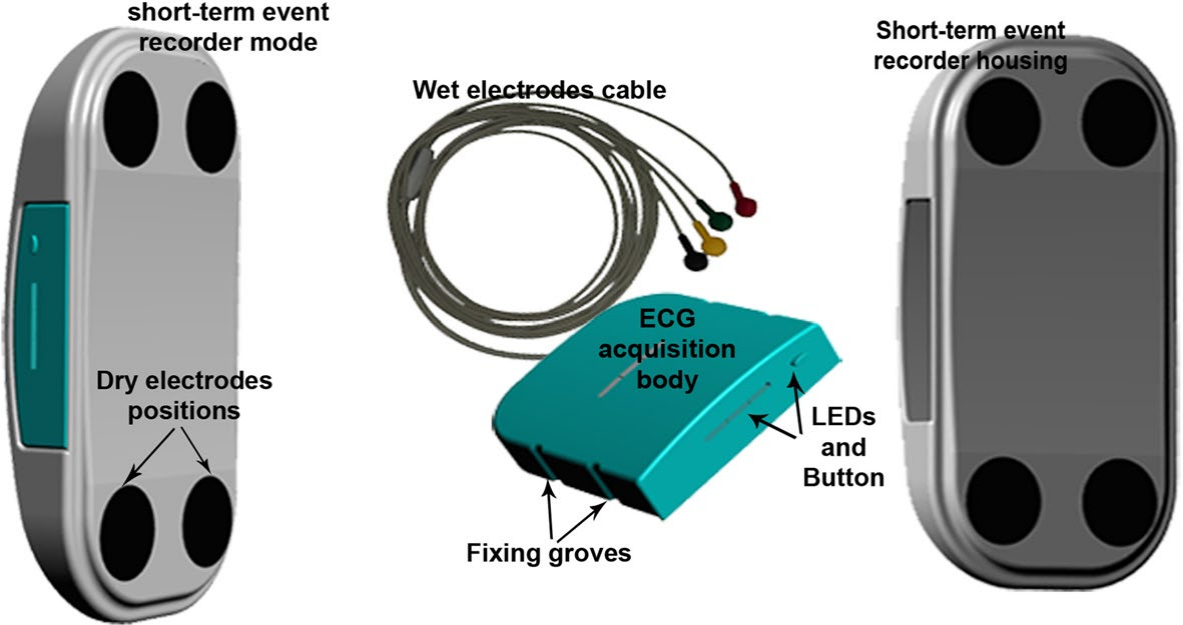
Shows the principal components and mechanical design of proposed ECG device, where short-term post-event recorder is enabled b inserting the ECG acquisition body in the short-term post-event recorder housing. Holter or long-term recording mode is enabled by connecting the cable of wet adhesive electrodes to the ECG acquisition body

Mechanical design of the ECG device presented in this paper is shown in Fig. 2. It mainly consists of a short-term post-event recorder body, and a long-term recorder body which is also the main ECG acquisition module. The separation of these two main parts allows the device to work in two independent modes: short-term post-event recording and long-term Event/Holter recording mode.

Consequently, patients can always carry the device around in their pockets and in case of typical testing, chest pain, or other arrhythmia symptoms, they can apply the device to the chest area and start recording three ECG channels, sensed by dry electrodes, without any preparation and wires. For this reason, the event recorder housing is provided with four dry electrodes positioned in the corners of an imaginary rectangular shape whose vertices are drawn on the slightly curved housing.

The short-term post-event recorder housing has inner jumper pins that are responsible for detecting the working mode of the device. Hence, when an ECG acquisition body is inserted into the short-term post-event recorder housing, the device activates a short-term post-event recorder mode and the ECG signal recording is performed using dry electrodes. In this mode, ECG main acquisition module is locked in the event short-term housing. This is achieved by embedding several latching blocks in the short-term post-event recorder housing and when the ECG acquisition body is inserted, they fix on several latch grooves on the side faces of acquisition body.

In order to run the device in the long-term recording mode, a user can easily extract the acquisition body using finger nails and attach the wet adhesive electrodes cable to start recording three standard ECG channels. For this reason, two slits between the ECG acquisition body and the short-term cover housing are left.

### Dry and wet electrodes

The main problem associated with long-term ECG signal recording is signal quality vs. noise and motion artifacts. Signal quality is significantly affected by electrode-skin impedance and by electrode’s stability on the subject’s chest. For this reason, it is important to apply the right type of electrodes that last for a long time and are able to record reliable ECG signal according to the selected working mode.

The stability of Ag/AgCl electrodes, along with their low electrode-skin impedance, makes them the most common and favored electrodes for ECG measurements. These electrodes are non-polarizable electrodes, so the charge can cross the electrolytic gel which is used to facilitate the electrochemical reactions and to reduce electrode-skin interface impedance. Thus, they are associated with low electrode-skin impedance, low noise and low motion artifact [12]. For these reasons, disposable wet Ag/AgCl electrodes are used for long-term recording and electrodes’ snap connectors’ cable is provided with the device.

On the other hand, short term event recording requires electrodes that can last for a long time and need minimal preparation. Dry electrodes are the best choice for short-term fast event recording, mainly because they don’t need any prior preparation. The materials from which the dry electrodes are made are more durable than Ag/AgCl electrodes; therefore, they do not need to be changed after recording [12, 15].

However, they are polarized electrodes and their skin-electrode impedance is higher in the frequency band of ECG signal. Authors in [12, 15, 16] compared the skin-impedance of different types of electrodes made of different materials. The results of their study showed that Orbital dry electrodes give superior performance as opposed to other dry electrodes in terms of skin-electrode impedance. Furthermore, orbital electrodes have pins or spikes on their contact surface that support the strong attachment of electrodes to skin since they penetrate the highly resistant skin stratum corneum layer. This helps to reduce the skin-electrode impedance, and stabilize the device body on the subject’s chest, which influences positively the recorded ECG signal quality. Therefore, we used these dry electrodes [17] for short-term recording. In order to overcome the skin-electrode impedance difference between dry and wet electrodes, we control the resistance at the instrumentation amplifier input in the electrodes analog front end. Thus, higher input impedance is used when event mode is activated to record ECG with dry electrodes. This helps to minimize the loading effect and ensures signal amplitude consistency in both modes [18].

Another important issue is the distance between electrodes and its effect on signal amplitude. The chest size has great impact on the signal recorded in the short-term even mode because the distance between the electrodes is fixed (14 × 7 cm) for all chest sizes. To resolve this issue, a special step, in the analysis pipeline of the signals, is added to extract reference templates and then use them in the analysis of the signals, as will be discussed in more details later in this paper.

### ECG acquisition module

**Fig. 3:**
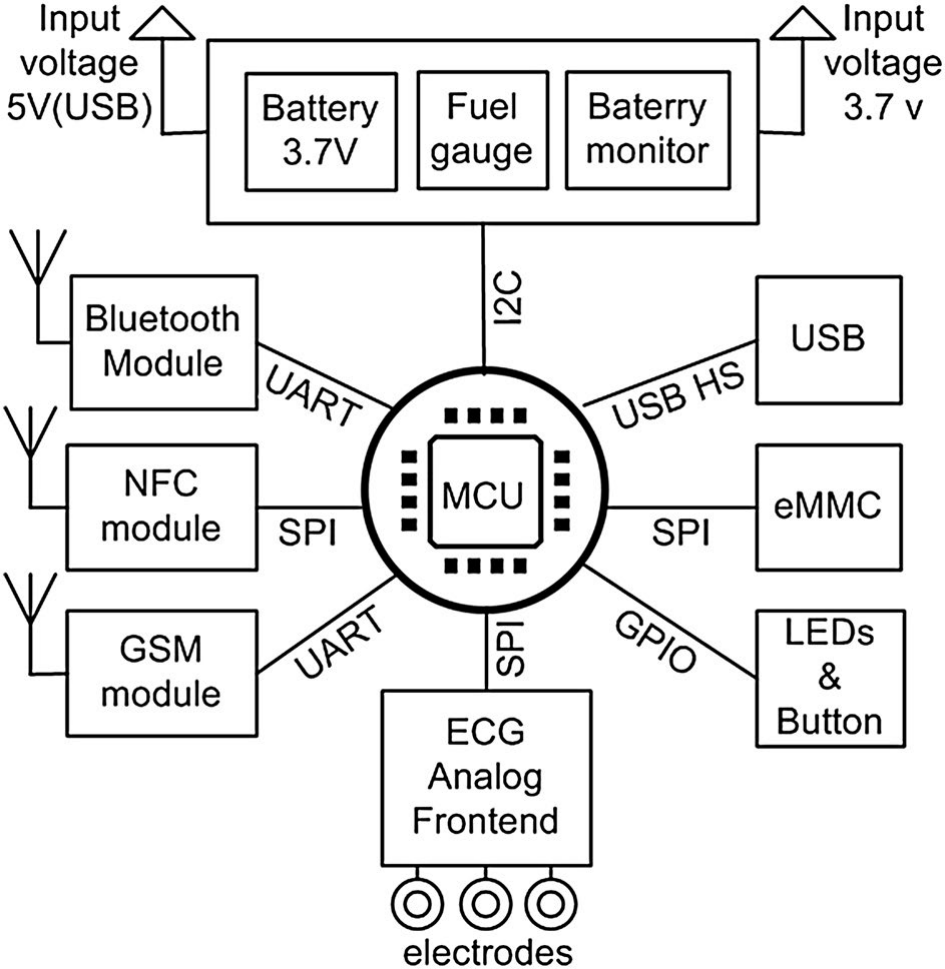
ECG acquisition module architecture

The block diagram of the ECG acquisition module is shown in Fig. 3. All components are embedded in the ECG device except for electrodes and interconnections. The first and most important component is the ECG signal analog front end. We used an on-chip device presented in [19]. This chip is designed and tested following the AAMI EC11 standard to simplify the task of acquiring and ensuring the quality of ECG signals. Wherein, it has amplifiers and analog to digital converters (ADC) able to provide up to five ECG channels in low power operation mode of 15 mW for three leads. Additionally, it has an embedded right leg driver logic which we set and used for lead-off detection and noise rejection which helps solve the problems caused by broken lead occurrence, or poor electrode-skin contact and to eliminate interference noise by actively canceling the interference [19]. The on-chip device was set to work at 19-bit level in 2 KHz data rate, which is later downsampled to 250 Hz. Serial Peripheral Interface (SPI) communication is implemented to transmit data and control commands between the on-chip device and host processor.

The ECG module also has a host processor (MCU), internal memory (eMMC) able to save patients’ information, and three leads recordings up to 7 days, a lithium battery 3.7 V along with its charging facilities (battery charger chip and fuel gauge), a Bluetooth transmission module, a GSM transmission module, one button and indicating Light-emitting diodes (LEDs), a near field communication (NFC) module, and, finally, a USB I/O port for charging, testing, and wired file transmission. Universal asynchronous receiver/transmitter (UART) communication is implemented to to enable the communication between the GSM and the MCU modules.

The usage of NFC module for the telemedicine medical devices was presented in [20–22]. The near field communication module addition makes the procedure of event recording, based on mobile phones, autonomous, easy-to-use and instant. The NFC module is embedded in the proposed device with an Radio-frequency identification (RFID) tag and a field detector, and is set to work in passive mode. The automatic pairing of a smart phone and an ECG device is activated when a patient moves the back of the smart phone toward the back of an ECG recorder. Thus, when the field detector detects the mobile phone NFC field, it activates a microcontroller by raising interrupt that starts the recording workflow. Simultaneously, the mobile phone reads the connection information from the RFID tag to launch a smart phone application and to establish a Bluetooth pairing with the ECG device.

### Mobile application

Medical data exchange between experts and patients is enabled using two smart phone applications built as a part of the telemedicine platform proposed in this paper.

The first application is the patient’s, which was built to help patients record the ECG signal and exchange messages and medical information, such as symptoms, with health centres and physicians. This information will be associated with a recorded signal when it is sent to the algorithms/storage server.

The second application is the expert application, which allows an expert to record and monitor ECG signals in real time, as well as to view, and analyse sent recordings, using algorithms running on the cloud server. Beside patient’s signal viewing and analysis, experts can exchange medical advices, feedback, and messages with patients, if necessary. Additional services were implemented to allow medical experts to exchange intervals of ECG signal and medical knowledge or opinions with other experts who are more experienced in the field of arrhythmology.

**Fig. 4:**
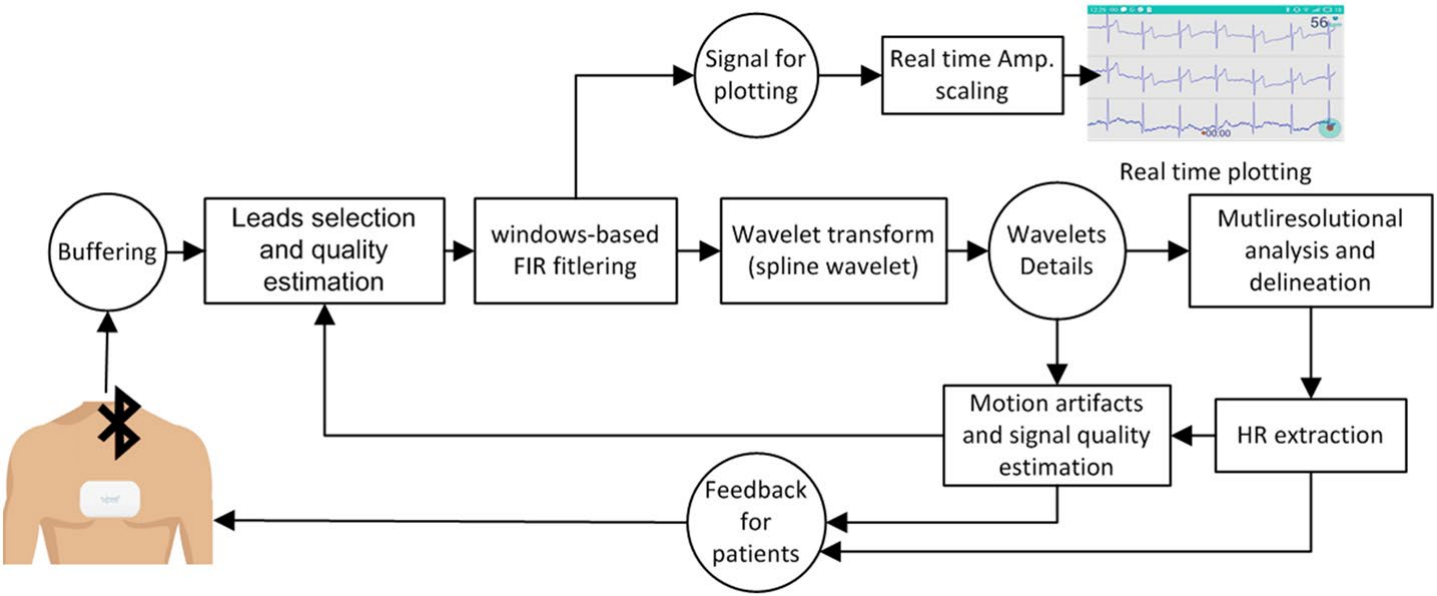
The flowchart of ECG signal processing pipeline implemented on smart phone applications

On both applications, a library for real time event ECG signal processing and basic analysis was implemented, which allows signal plotting on mobile monitors, and provides heart rate and signal quality information as feedback to patients. The basic flowchart of the real time processing library is shown in Fig. 4.

Hence, the received signal is buffered in a 1-s buffer, and then the signal is filtered from both baseline wandering and high frequencies noises, such as EMG noises and network interference. Its amplitude is then scaled in real time to ensure that its maximum and minimum values fit the smart phone display. A spline wavelet transform is also applied to delineate the ECG signal and, consequently, extract the heart rate. For this reason, the state of art multi-resolutional approach, presented in [23], was used. Wavelet transform details at scale $2^2$ , along with the heart rate extracted in the delineation process were used to estimate motion artifacts and EMG noise. Difference between the original wavelet details and the aligned averaged details signal for QRS complexes is used to define signal quality at each interval in the ECG signal. This approach is presented in [24]; however, we used wavelet details at scale $2^2$ instead of an ECG signal, because most of the energy of QRS complexes lies in this scale [23, 25]. Information about estimated leads quality as well extracted heart rate are shown and updated in real time.

The mobile phone applications are native mobile applications and support both operating systems IOS and Android. Processing library is written in C language and wrapped to be used in Java for the Android application and objective C for the IOS application. Bluetooth connection was used to enable real time plotting of the received signals from the paired device. Additional pages are designed for the device, patient, and patient parameter setting.

### Algorithms and offline analysis

**Fig. 5:**
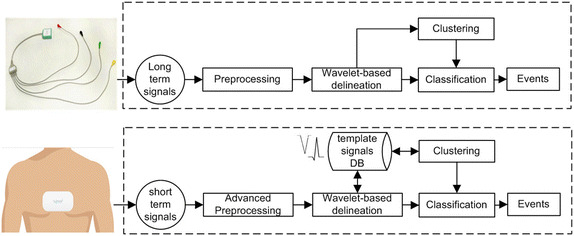
The flowchart of ECG signal analysis for both short-term and long-term modes

The next step, after sending signals to the algorithms/storage server, is to process the signals and provide automatic analysis report associated with the signals. The flowchart of our automatic analysis for long-term signals, as well as for short-term signals, is shown in Fig. 5. Both analysis workflows share the main components of preprocessing, feature extraction and delineation, and, finally, the arrhythmia detection (classification and clustering).

However, analysis workflow of ECG signals, recorded by the proposed device, changes according to the recording mode due to different leads lengths, and different electrodes positions and types. The short-term post-event signals recorded using dry electrodes are more difficult to be analysed, because of the lack of dominant beat reliability caused by small beats number recorded in this mode. Additionally, the positioning of event recorder on patient’s chest has a great impact on the ECG waves’ morphology and polarity in the short-term post-event recording mode. This is due to different cardiac muscle positions and different axes [5].

So, the proposed solution requires templates to be built for each patient when patient starts using the short-term post-event recorder. The templates are built by testing relatively different positions on patient’s chest the first time they use the device. The device placement that provides the best signal quality will be used and recordings from that position will become the source of normal QRS templates that are saved and used moving forward. The tested positions are around specific position predefined as the standard device placement position for this device design. This is discussed in details later in evaluation section.

On the other hand, when long-term holter signals are recorded there is no need to use any predefined templates in the analysis and interpretation pipeline. This is because average beat could be dependably computed from the large number of recorded beats (central limit theory). Average beat could be used later in several steps in the analysis pipeline; to estimate signal quality and to find the fluctuations of the beats’ morphology.

Therefore, the first stage of the both modes signals analysis pipeline is the preprocessing stage. Firslty, ECG signal is filtered from both baseline wandering and network interference using an FIR filter with reduced number of Taps presented in [26], while high frequency and EMG noise was filtered using an FIR filtering according to the specifications and recommendations of bandwidth used in filtering [27].

Afterwards, the quality of each lead was estimated using more sophisticated time-invariant algorithm than that used for real time processing. This algorithm is used to estimate the signal quality vs. motion artifacts and baseline artifacts and high frequency EMG noises [28]. Subsequently, the leads quality estimation is used in leads selection logic to use one, two, or all three leads for delineation, clustering, and classification stages. The right selection of leads to be used in the analysis is important since it affects ECG waves’ delineation and beats classification [29, 30].

Next step is to apply spline wavelet transform to delineate ECG waves. The same algorithm used in mobile-based ECG processing was used for this purpose [23]. Then, a combination of the delineation results was done using the signal quality representation of each lead as in [31]. This approach reduces the negative impact of noisy intervals on delineation results. Additionally, the combination of single-lead delineation results increases the positive predictive values and the sensitivity values of overall QRS detections, by taking advantage of the three leads presence. Combination is achieved using several criteria. For instance, when signal quality, estimated over time for each lead, worsens for some leads, then other leads with the better signal quality should be used. Another example is when a beat is detected on one lead while is absent on others. This is considered a false predictive beat.

Clustering algorithm is then built to cluster the detected beats into forms which are used in the classification stage of these beats. Wherein, each ECG beat was encoded in vector of 6 digits of KLT transform coefficients extracted as described in [32, 33], and two more digits from RR intervals as used in [34] are added. These vectors are then normalized and K-means algorithm was used to cluster the ECG beats. Finally, classification algorithm, presented in [35], was used to find the class of extracted beats. For short-term signals, all beats from the cluster whose morphology is similar to the predefined normal beat morphology are associated to the normal class after considering their heart rate features.

All beat annotations are mapped during the classification process into the set N, V, S, Q (corresponding to normal, ventricular ectopic, supraventricular ectopic, unknown). Finally, a report with clusters’ morphological forms, delineation statistics, along with intervals of interest is introduced to physicians for detailed analysis.

Calibration of the patients’ templates is of paramount importance. It should be taken into consideration by physicians because of the acquired template changes during the lifespan of all patients, especially the younger ones. The templates can be changed easily using the mobile phone applications by physicians or by patients themselves. Patients, who would use the device for long periods or before and after some circumstances that could change the templates morphology, must recalibrate the morphology and the analysis parameters of their personal ECG recordings.

Three groups of customizable parameters—pediatrics, adults, and special—are used as default analysis parameters. The first group, or the pediatric group contains normal ECG parameters for children aged 0–16 years divided into several age groups [36, 37]. The second group is the adult group. However, all parameters for groups can be also customized according to each patient’s case in a special group of parameters. For instance, patients with Acquired Heart Block due to surgery or medication, or with congenital Heart Block that developed after birth, should have customized analysis parameters which must be controlled by physicians, and fluctuations from those parameters should be considered as abnormal changes. Another example is in sport medicine, where athletes have special parameters that depend on their sports, special conditions, and age [38, 39]. A special set of parameters should be used to handle any special situation.

Therefore, we used a patient-parameters database that contain used analysis parameters along with the template ECG wave for each patient. The patient-parameters database is editable and must be calibrated by physicians according to patients’ changing conditions.

All algorithms were designed firstly using MATLAB and Python Packages. They are then ported to C programming language and wrapped in python back-end so that the communication between the cloud-based web application and the wrapped algorithms is done using REST services implemented within Django REST framework.

**Fig. 6:**
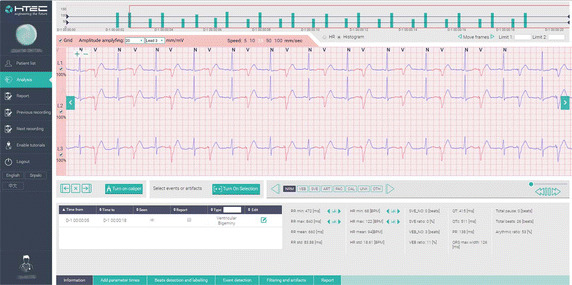
Screen-shot of the web analysis platform. Automatic analysis results are seen in the *bottom part*, while the signal is shown with *colors* annotating the beats classification. Physicians have an access to their patients recordings so they can confirm the automatic analysis results and follow their status

Screenshot of the front-end web application is shown in Fig. 6. Different beats’ classes are shown by plotting beats in corresponding standard colors. The algorithm-based interpretation (Ventricular Bigenminy) is shown in table to be confirmed by physician.

## Evaluation and results

Long-term ECG signals, recorded by the proposed device, are standard holter signals recorded using wet electrodes and the long-term mode itself is not the novelty of this paper. For this reason, only validation procedures of short-term patient-activated event signals, recorded by the means of dry electrodes, are presented in this context.

To evaluate the short-term post-event recorder design introduced in this paper, a clinical study was conducted. A total population of 391 patients was tested in the evaluation process, 40 volunteers and 351 patients with non-significant cardiac issues. The average age of validation population, included in this study, was $26.90 \pm 19.32$ (4–80 years). The genders percentages of tested patients are 60.86% or 238 males, and 39.13% or 153 females. The adults (age > 16) percentage is 52.94% or 206 adults, while the percentage of children (age ≤ 16) is 47.05% or 184. The evaluation procedures were divided into two phases; prevalidation and validation.

The purpose of the prevalidation process was to find the best placement of short-term post-event recorder on subjects’ chest. Total of 60 participants were selected in the prevalidation procedures, while the other evaluation procedures were finished with the residue validation population 331 participants.

In both procedures, the main tested body positions were supine, sitting, and standing positions. Patients recorded their ECG by themselves, but all recordings were performed under the supervision of medical professionals. Measurements were done without skin preparation such as shaving or adding conductive gel on the skin surface, and signal recording was performed immediately after placing the device body on the subject’s chest. The whole study was carried out following the rules of “The 1975 Declaration of Helsinki” [40]. All the evaluation procedures were approved by the Belgrade University Children’s hospital ethics committee, and the participants’ informed consent was given before the experiment.

### Device placement versus signal quality

**Fig. 7:**
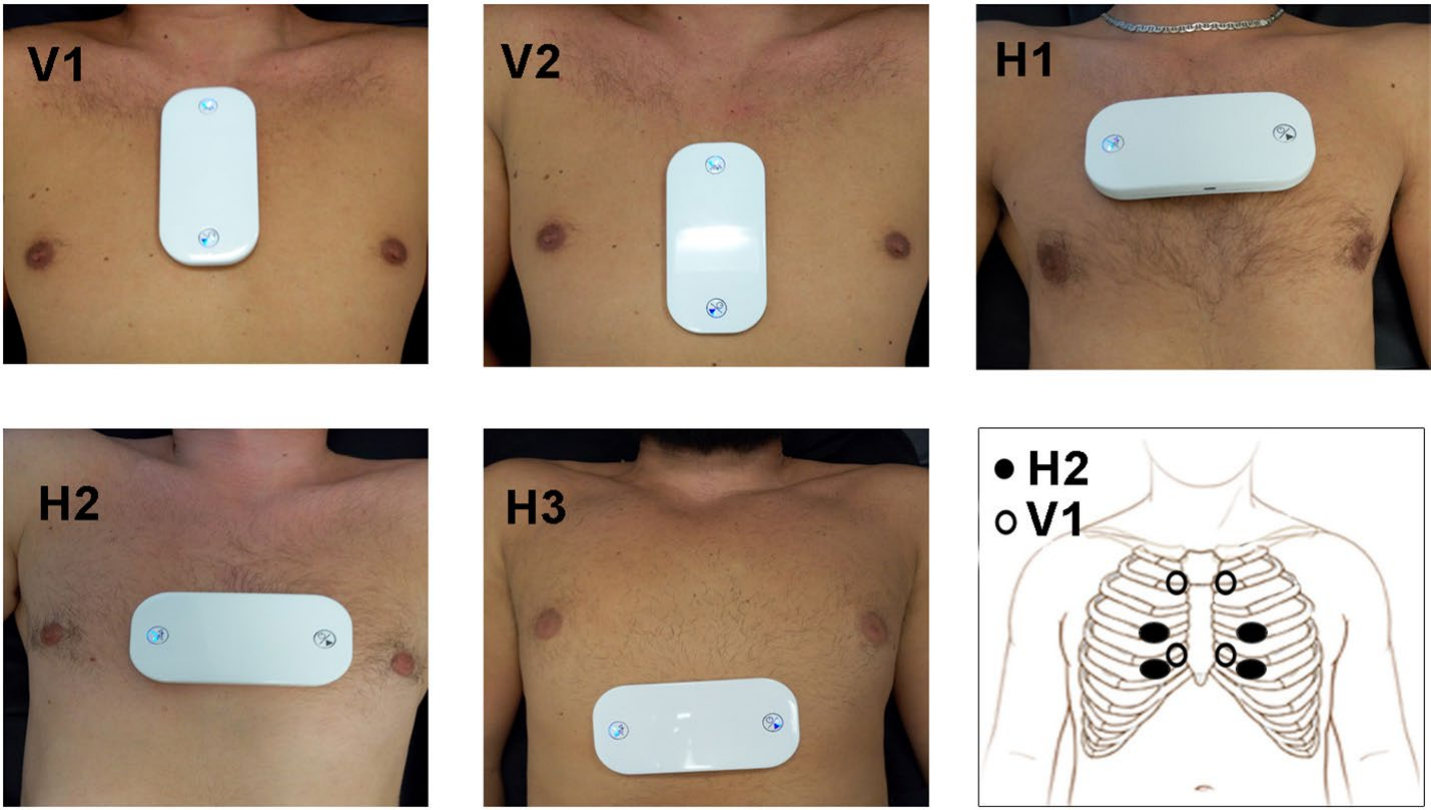
Tested device placement; two vertical (V1–V2), three horizontal (H1–H3), and finally the corresponding positions of electrodes of H2, V1 placement on human chest ribs

In the prevalidation phase, our goal was to find the best placement at which three most different leads are sensed. This is important for physician because leads morphological difference reflects the heart muscle electrical activity from different angles [6, 8, 41]. For this reason, signals of 20 s length were recorded using the proposed short-term post-event recorder with different placements on each patient’s rib cage. The tested placements during the prevalidation phase are illustrated in Fig. 7.

Afterwards, two specialized cardiologists were asked to estimate signal quality for the analysis of the three channels recoded using dry electrodes. They went through the signals and annotated them in terms of signal quality and clinical acceptability. Signal quality refers to the presence of EMG noise, motion artifacts, and baseline wandering, while clinical acceptability refers to the presence of all PQRST waves, narrowness of QRS complex, and suitable R/T amplitude ratio. Experts were asked to give their estimate from 1 to 5, where 1 stands for unacceptable signal for analysis and 5 stands for high-quality signal, suitable for interpretation.

At the end of prevalidation process, position H2 gives the best results and was the best placement with good quality and different ECG channels morphology. This applies to a subset of the tested population which includes both adults ($age > 16$ years) and children (age $\le$ 16 years) with rib cage size allowing this placement. On the other hand, position V1 gives better results for children whose chest size doesn’t enable recording in position H2.

**Table 1:** Signal quality and clinical acceptability for selected placements H1, V1

Population	Age range	Signal quality	V1	H1
Adults	17–80	4.17 ± 0.30	0	30
Children	4–16	4.10 ± 0.28	6	24
Both	4–80	4.13 ± 0.20	6	54

The average signal quality annotated by experts of the signals at the selected positions, V1 and H1, was quantified per age group and presented in Table 1. Signal quality was presented with a standard error computed with a confidence interval of 95%.

Device placement illustration, according to age category is stored in the smart phone application. Instructions to help patients to find the best placement on chest, and to explain the correct usage of our device, were included in our smart phone application.

### Correlation with ECG golden standard leads

In order to evaluate the quality of ECG signals recorded by our short-term post-event recorder at the selected positions, we compared them to the golden standard 12 ECG leads. The correlation coefficient check was examined to understand the possible distortions caused by the usage of loose dry electrodes. Additionally, it was intended to find the maximum correlated lead from the golden standard 12 leads ECG to each lead from event recorder device.

The correlation coefficient between the recorded three leads, using dry electrodes, and ECG signals recorded simultaneously using 12 leads gold standard ECG (SCHILLER CARDIOVIT CS-200 Office System) was computed.

**Table 2:** The average correlation values of short-term post-event recorder Leads (L1–L3) and corresponding ECG Golden standard leads (V1–V3)

Compared leads	Correlation coefficients
V1–L1	0.888
V2–L2	0.8930
V3–L3	0.929

After analysing a sample of 100 recordings, of 20 sec length, from the validation population signals, we found that golden standard precordial leads (V1, V2, and V3) were the best match with ECG leads recorded by our short-term post-event recorder, since they show a high correlation with our short-term leads, recorded using dry electrodes. The computed correlation coefficients with these leads and our leads are presented in Table 2. Thus, we call the leads recorded by our short-term post-event recorder modified V1, V2, and V3 leads. Consequently the usage of short-term leads should be equivalent to the usage of golden standard leads in terms in applicability and reliability in arrhythmias detection.

**Fig. 8:**
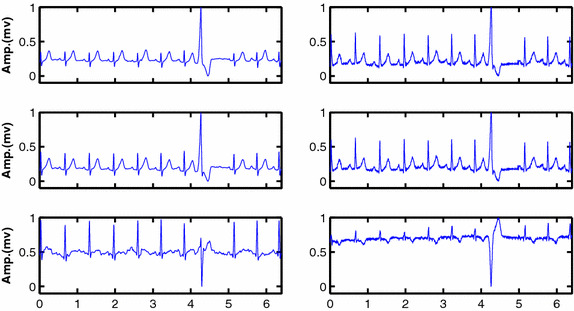
Shows short-term post-event signals L1–L3 (*left column*) versus golden standard channels V1–V3 (*right column*). The morphological variability could be noticed among leads recorded using short-term post-event recorder as well as golden standard leads

Figure 8 shows three leads of ECG signals recorded using our proposed design with dry electrodes and corresponding leads of the ECG golden standard device. The most important point to highlight and deduce from this figure is that the short-term post-event mode of the proposed device was able to record three different leads that represent the heart muscle electrical activity from different angles, exactly as the golden standard ECG recorder did. Another point that could be deduced from this figure is the equivalent signal quality regardless of different electrodes types used in each recorder.

### Peer review of clinical acceptability

Quality of signal is not only restricted to the cleanness of signal from artifacts and noises. The ability to do a detailed interpretation of ECG signals is also a paramount necessity. This includes the presence of ECG main waves (P, Q, R, S, and T), as well as suitable morphology and amplitude for them that allow experts and algorithms to measure the width and amplitude variation of ECG waves. For instance, the QRS complex should be tall and narrow (recommended amplitude >0.5 mV, but not biphasic), while T amplitude should be relatively smaller than the R wave [29, 30]. Such details have great impact on both diagnosis potential and, consequently, on automatic analysis. This is reflected in the performance of different algorithms for automatic delineation and analysis. To translate this into statistical data, we use two criteria to evaluate the the recorded signals acceptability for interpretation; expert-based and algorithm-based.

Firstly, we followed a peer review process to evaluate signal clinical acceptability. So, we presented three leads recorded by our device, as well as the three most correlated leads recorded simultaneously from the golden standard 12 leads ECG device, to two experts without providing them with information about signals’ origin. This was done for a sample of total 100 recordings. Experts were asked to annotate each set as valid or not valid for detailed analysis. For this reason, doctors went through the two sets A and B for each of three leads and gave their opinion as A, B, AB, none.

**Table 3:** Results of peer review of event and best match leads from golden standard ECG

Recording device	Clinical acceptability	
	Valid	Invalid
Short-term recorder	96	4
Golden standard ECG	98	2
Both devices	95	1

Results of this survey are presented in Table 3. Presented results show that the short-term post-event ECG signals, recorded using dry electrodes, have comparable diagnosis potential to the ECG 12 leads golden standard and could be used in arrhythmia detection.

Afterwards, we tested the hypothesis that the validity ratio of signals, recorded with short-term mode of the proposed device $P_e$, is equivalent to the validity ratio of signals recorded using the golden standard ECG recorder $P_g$. With a confidence interval of 95%, we found that standard error of the tested hypothesis is 0.829 and P value is 0.796. This leads us to accept the null hypothesis that both ratios are equivalent, and that short term signals could be used in similar way to the golden standard signals in heart rate variability analysis.

We found during this validation phase that in case of consistent pressure aimed to force the electrode against the subject’s skin, the signal quality of our leads, in terms of EMG noise and motion artifacts, was corresponding to standard ECG leads annotated by experts as the best match with our leads. Nonetheless, corresponding standard ECG leads signal quality in terms of baseline wandering was better than our leads, recorded by our device. Finally, 99% of tested patients succeeded in accomplishing a transmission test after following the instructions stored in the mobile phone application.

### Accuracy evaluation for heart rate detection

In order to examine the quality of recorded ECG signals in terms of fidelity in recording suitable ECG waves, we evaluate the performance of the automatic delineator.Both short-term post-event recorder leads, and the corresponding best matched three leads from the golden standard 12 leads ECG were tested. A sample of 100 recordings was used in this phase. Each recording contained 6 leads, three leads of each device. Recordings were done simultaneously using both devices and each was of 20 s length.

**Table 4:** QRS complex delineation results on both short term event leads and best matched three standard ECG leads event

Leads	Event recorder				Golden standard			
	L1	L2	L3	L1–L3	V1	V2	V3	V1–V3
PPV.%	93.18	96.56	95.27	99.07	98.04	99.14	97.22	99.34
Sens.%	97.55	99.61	98.76	99.23	99.13	99.87	99.83	99.87

Two expert annotators delineated the QRS complex independently, and their delineation was considered as the golden standard delineation for comparison. Afterwards, the delineation algorithm presented in [23] was used to detect QRS complexes automatically. Sensitivity and positive predictive value for QRS complex detection, after comparison to expert manual annotations, were computed and presented in Table 4.

Signals recorded using dry electrodes obtained a positive predictive value of 99.07% ,when a combination of single lead delineation results is used as we mention in algorithms section, compared to 99.34% from the corresponding leads from the golden standard ECG. These results show that automatic delineation algorithms’ performance is equivalent for short-term post-event recorder signals as well as for ECG golden standard recorder. Consequently, the QRS complexes could be dependably detected and used for heart rate variability analysis, including Atrial Fibrillation detection, in the ECG signals recorded using short-term post-event recorder.

### Noise influence on heart rate accuracy

To check the signal quality in terms of clinical acceptability for heart rate analysis, we calculate the percentage of detected beats on each lead, which were also detected on all leads.

This metric was used and presented in [42, 43]. It indicates the clinical quality of ECG channels in terms of resistance to noises and motion artifacts by measuring the performance of automatic QRS delineation on all leads. Since beats detection in high-quality signals is more accurate on all leads, there are less isolated beats that are detected erroneously by algorithms on each lead separately. The aforementioned state of the art delineator was used to detect QRS waves in 400 leads of post-event short-term recorder and in the corresponding leads from the 12 leads golden standard ECG recorder.

**Fig. 9:**
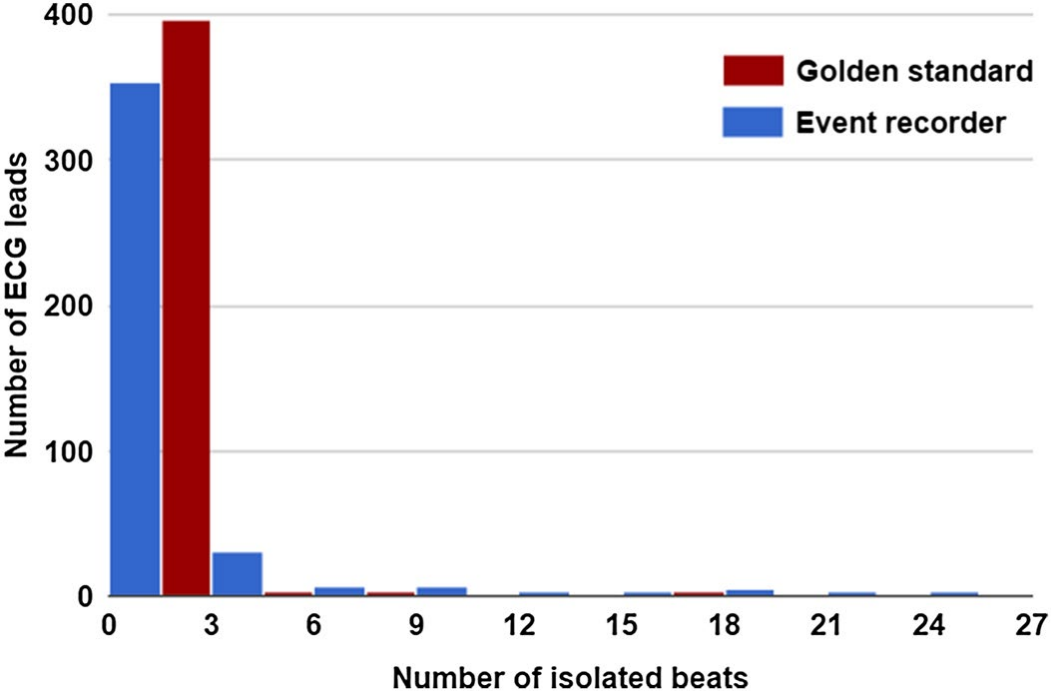
Histogram of isolated beats or detected on each lead that are not detected on all leads

Results are shown in Fig. 9 and they indicate very good performance for automatic delineator with our leads, as with the corresponding golden standard ECG leads. This is an indication of equivalent signals quality and applicability for hear rate detection and subsequent arhythmias analysis.

To translate the presented results from Fig. 9 into statistical measure, we test the mean difference of the paired ECG delineation results (isolated beats number). The tested hypothesis is that the difference of isolated beats numbers of delineated leads, recorded simultaneously using the short-term mode of the proposed device and the golden standard ECG recorder, is greater than zero. With a confidence interval of 95%, we found that standard error of the tested hypothesis is 4.52 and P value is 0.99. This leads us to reject the null hypothesis and to accept the alternative that isolated beats number ratios are equivalent.

### Comparison with the available commercial solutions

**Table 5:** Features comparison with similar available commercial solutions

Name	Unit description	Channels number	Applications	Electrodes types	Recording place	Transmission methods
Alivecor system and ECG check	Smartphone protective case	Single channel	Short-term post-event tests	Dry	Fingers	FM
EPI mini	Independent unit	Single channel	Short-term post-event tests	Dry	Fingers	Bluetooth
eMotion ECG	Continuous wearing, cable set, wearable belt or fastfix	Single or three channels	Real time monitoring, holter monitoring	Wet adhesive	Chest	Bluetooth
NUVANT mobile	Continuous wearing and measuring	Single channel	Real time and holter monitoring	Wet adhesive	Chest	Bluetooth
Ambulatory ECG	Independent unit	Single channel	Post event monitoring, patient activated	Dry	Chest	Bluetooth
Omron	Independent unit	Single channel	Post event short-term monitoring, patient activated	Dry	Chest	Bluetooth
Body guardian verit	Independent unit	Three channels	Long-term and Holter monitoring	Wet adhesive	Chest	Bluetooth and cable
IEM beam	Independent unit	Three channels	Short-term loop/event recorder, record up to 3 min	Wet adhesive and dry electrodes	Chest	Bluetooth
Proposed design	Independent unit provided with NFC module for fast activation	Three channels	Long-term event and holter monitoring, post event monitoring short-term monitoring	Wet adhesive and dry electrodes	Chest	Bluetooth, GSM and cable

Finally, we compare features of the proposed device with other similar available commercial solutions. Table 5 explains the features differences of the proposed device compared to some known solutions.

The most important advance of the proposed design, compared to some of those commercial solutions, is the reliability of recorded ECG leads for deep analysis. This is achieved by using the appropriate electrodes number and types (dry and wet) with hardware customized for each of those types. Devices with a single lead could not be considered confident for deep ECG signal analysis [44]. On the other hand, the usage of wearable fashion to record ECG signals is still subject of debate since signals recorded using this approach suffer from motion artifacts and noises that reduce the clinical acceptability of such signals [45].

Therefore, we stated in this paper that reliable long-term recording, as well as fast reliable short-term recording, could be achieved using both dry electrodes and wet adhesive electrodes. To increase the reliability and acceptability of recorded signals analysis we proposed a customized algorithmic approach dealing with signals depending on the used electrodes, and on the patients special ECG templates in the short-term mode.

The usage of an NFC module reduces the time needed to start short-term post-event recording, which is a very important issue in short-term post-event recording.

Finally, the hardware costs of single device, operating as we proposed, are significantly smaller than the costs of two devices each operating in separating recording mode (short-term post-event and long-term holter).

## Conclusions

We present a multi-purpose ECG telemedicine system that can operate in different working modes. The simple design and the usage of dry electrodes for short-term post-event recording and wet adhesive for holter long-term mode, allows laypersons to record reliable signals according to physician’s recommendations in each of these modes.

The reliability of three post-event short-term ECG leads with direct symptom-rhythm correlation is the major advantage of the short-term post-event mode.This is achieved by providing solutions to the drawbacks of already available devices while focusing on maintaining of the recorded signals’ reliability.

The evaluation of proposed novel design of event recorder with dry electrodes, showed that ECG signals of 96% of participants, who finished the recording and transmission, have the diagnosis potential to be used in arrhythmia detection for different age groups.

## Abbreviations

SNR: signal-to-noise ratio
EMG: electromyography
KLT: Karhunen-Loève Theorem
GSM: global system for mobile communication
GPRS: General Packet Radio Service
Sens: sensitivity
PPV: positive predictivity value
eMMC: embedded MultiMediaCard
Ag–AgCl: silver chloride electrode
AAMI: Association for the Advancement of Medical Instrumentation
FIR: finite impulse filter
MCU: microcontroller unit
LED: light-emitting diode
GPIO: general purpose input/output
USB HS: high-speed universal serial bus
I2C: interintegrated circuit
I/O: input/output
L: lead
